# Feasibility and short-term outcomes of bioadaptor in bifurcation intervention using culotte technique: intravascular ultrasound analysis from a single-center experience

**DOI:** 10.1186/s44348-026-00083-8

**Published:** 2026-07-21

**Authors:** Van Hoang, Giang Tra Tran, Dung Tran Ngoc, Duong Nguyen Dang, Dong Tran Van, Cuong Pham Hung, Tien Tran Dinh

**Affiliations:** 1 Department of Interventional Cardiology, Hanoi Heart Hospital, Hanoi, Vietnam; 2 International Department, Hanoi Heart Hospital, Hanoi, Vietnam; 3 Department of Internal Medicine, Hanoi Heart Hospital, Hanoi, Vietnam

**Keywords:** Bifurcation percutaneous coronary interventions, Coronary bioadaptor, Culotte technique, DynamX, Intravascular ultrasound, Two-stent strategy

## Abstract

**Background:**

The DynamX bioadaptor is a novel coronary implant with three helical strands that unlock and separate to provide dynamic vessel support restoring natural vessel function. Evidence in complex bifurcation lesions requiring planned two-stent strategies is limited. This analysis evaluated the feasibility and short-term outcomes of culotte bioadaptor implantation using intravascular ultrasound (IVUS).

**Methods:**

Thirteen consecutive patients underwent bifurcation percutaneous coronary intervention using culotte technique with DynamX bioadaptor between February and October 2025. All procedures followed a standardized culotte protocol with lesion preparation, proximal optimization technique, rewiring, and final kissing balloon inflation, with IVUS used to assess bioadaptor expansion, apposition, and structural integrity.

**Results:**

Technical and procedural success was achieved in all patients (100%), with successful rewiring through bioadaptor struts in all attempts (26 of 26, 100%). IVUS demonstrated satisfactory expansion: main branch ostial expansion 94.9% ± 10.6%, side branch ostial expansion 90.2% ± 17.5%, and proximal expansion ratio 0.95 ± 0.16. No strut fractures or malapposition occurred.

**Conclusions:**

Early experience with bioadaptor implantation in culotte bifurcation stenting demonstrates feasibility, with favorable acute IVUS findings in selected patients.

## Background

Coronary bifurcation lesions account for approximately 15% to 20% of all percutaneous coronary interventions (PCIs) and remain among the most technically challenging lesion subsets [1]. Despite advances in stent platforms and procedural techniques, bifurcation PCI continues to be associated with higher rates of procedural complications, restenosis, and stent thrombosis compared with non-bifurcation lesions [2, 3].

Although a provisional single-stent strategy is preferred for most bifurcation lesions, two-stent techniques are required in approximately 20% to 30% of true bifurcations, particularly when the side branch is large, diffusely diseased, or supplies substantial myocardium [4, 5]. Among two-stent approaches, the culotte technique provides comprehensive carina and side branch ostial coverage with favorable outcomes when combined with proximal optimization technique (POT) and final kissing balloon inflation (KBI) [6, 7].

Successful culotte stenting requires specific stent platform characteristics [8, 9]. These include thin strut thickness (< 80 µm) to minimize metal burden and flow disturbance [10], adequate flexibility for navigating tortuous anatomy [11], reliable rewirability through stent struts to enable distal cell crossing [12, 13], sufficient radial strength to resist recoil during high-pressure inflations [11], and broad expansion capacity to accommodate vessel size mismatch without strut fracture [14].

The DynamX bioadaptor (Elixir Medical Corp) is a novel coronary implant designed to provide acute drug-eluting stent–like performance while restoring long-term vessel physiology [15, 16]. The device consists of three 71-µm cobalt-chromium helical strands interconnected by bioresorbable polymer-coated uncaging elements that degrade over approximately 6 months, enabling strands to unlock, separate and adapt to vessel motion.

Clinical evidence demonstrates favorable outcomes. The BIOADAPTOR RCT study demonstrated noninferiority to contemporary zotarolimus-eluting stents at 12 months, with superior imaging endpoints including reduced late lumen loss and restoration of cyclic pulsatility [17]. At 2-year follow-up, the trial demonstrated a statistically significant 65% reduction in target lesion failure (TLF; 1.8% vs 5.5%, *P* = 0.044) [18]. The INFINITY-SWEDEHEART trial subsequently confirmed these findings, demonstrating 48% reduction in TLF (hazard ratio [HR], 0.52; 95% confidence interval [CI], 0.29–0.93; *P* = 0.027) in the landmark analysis from 6 months through 2 years [19].

The culotte technique requires sequential rewiring and creates double metal layers proximally, providing large mechanical stresses on the implant. The bioadaptor mechanism of action may offer long-term advantages in bifurcations which are associated with adverse shear stresses that promote neoatherosclerosis and late stent thrombosis [20, 21]. Preliminary evidence supporting this approach was reported in the ADAPT-CULOTTE study, describing double bioadaptors in culotte configuration [22], and the present analysis further evaluated the technical feasibility, expansion characteristics, and acute intravascular ultrasound (IVUS) outcomes of the bioadaptor in planned culotte bifurcation PCI.

Despite these results, the performance of the bioadaptor in complex bifurcation PCI using two-stent strategies remains undefined. This study reports the first systematic evaluation of bioadaptor feasibility in culotte bifurcation PCI using comprehensive IVUS analysis.

## Methods

### Ethics statement

The study protocol was approved by the Hanoi Heart Hospital Ethics Committee (No. 1099/BVT-GCNHĐĐĐ). All patients provided written informed consent prior to enrollment. The study was conducted in accordance with the Declaration of Helsinki.

### Study design and setting

Between February 2025 and October 2025, we prospectively enrolled 13 consecutive patients undergoing bifurcation PCI using the culotte technique with DynamX bioadaptor at the Department of Interventional Cardiology, Hanoi Heart Hospital.

### Study population

Inclusion criteria comprised symptomatic coronary artery disease with established indication for revascularization, de novo true bifurcation lesion classified as Medina 1,1,1, 1,0,1, or 0,1,1 morphology, side branch reference diameter ≥ 2.5 mm by visual estimation, side branch lesion length > 5 mm or presence of ostial disease, and absence of significant size mismatch between proximal main vessel and side branch. Exclusion criteria included cardiogenic shock, severe calcification requiring rotational atherectomy, and contraindication to dual antiplatelet therapy. Left main bifurcation lesions were not included in this initial feasibility cohort. The study was designed to evaluate bioadaptor performance in non–left-main true bifurcations treated with a standardized culotte protocol. Left main PCI was excluded because it represents a higher-risk anatomical subset with larger vessel caliber, greater myocardial territory at risk, and distinct sizing and optimization considerations.

### Procedural technique

All procedures were performed via transradial or transfemoral access using 6 F or 7 F guide catheters. Culotte stenting followed a standardized protocol: (1) dual-wire lesion preparation with balloon predilatation and IVUS before or immediately after predilatation; (2) first stent deployment in the more angulated branch using a bioadaptor sized 1:1 to the distal reference diameter, protruding 2 to 3 mm into the proximal main vessel; (3) first POT; (4) rewiring through stent struts with distal cell crossing and strut dilation using a 1.5- to 2.0-mm balloon; (5) second stent deployment in the main vessel; (6) second POT; (7) side branch rewiring; (8) final KBI with two noncompliant balloons sized 1:1; (9) final POT to restore circular geometry; and (10) final IVUS assessment of all segments.

### IVUS protocol

IVUS was performed using the AVVIGO Guidance System (Boston Scientific) with automated 0.5-mm/sec pullback. Quantitative IVUS analysis assessed minimal lumen area, stent area and expansion, stent apposition assessment with malapposition defined as > 200 µm separation between strut and vessel wall, assessment of strut layers in the proximal overlapping segment, coverage of the carina and side branch ostium, and evaluation for edge dissection or tissue prolapse. Qualitative analysis evaluated helical bioadaptor visibility, strut distribution, and structural abnormalities, including fracture or deformation.

### Study endpoints

The primary endpoint was technical success, defined as successful deployment of both bioadaptor stents with < 30% residual stenosis by visual assessment and TIMI grade flow 3 in both branches. Secondary endpoints included procedural success (technical success without in-hospital major adverse cardiac events [death, myocardial infarction, or urgent target vessel revascularization]), rewiring success rate through stent struts, final KBI success rate, IVUS-defined stent expansion and apposition, procedural complications, fluoroscopy time, and contrast volume.

Periprocedural myocardial infarction was assessed according to the Fourth Universal Definition of Myocardial Infarction [23]. High-sensitivity troponin T (hs-TnT), creatine kinase (CK), and CK-MB were reviewed when available. The local laboratory upper reference limit for hs-TnT was < 14 ng/L. In patients with elevated baseline hs-TnT, serial biomarker values were assessed to determine whether the baseline was stable, falling, or dynamically rising before PCI. Periprocedural myocardial infarction required biomarker elevation together with clinical, electrocardiographic, imaging, or angiographic evidence of new myocardial ischemia.

### Statistical analysis

Continuous variables are expressed as mean ± standard deviation or median with interquartile range depending on distribution. Categorical variables are presented as frequencies and percentages. Given the exploratory nature and limited sample size, formal statistical hypothesis testing was not performed.

## Results

### Baseline characteristics

Thirteen patients (mean age, 60.3 ± 9.8 years; 11 male patients, 84.6%) were enrolled. Mean body mass index was 22.4 ± 3.0 kg/m^2^. Clinical presentation comprised chronic coronary syndrome in eight patients (61.5%), non–ST-segment elevation myocardial infarction in three patients (23.1%), and unstable angina in one patient (7.7%). Cardiovascular risk factors included current or former smoking in nine patients (69.2%), diabetes mellitus in five patients (38.5%), and hypertension in six patients (46.2%). Mean estimated glomerular filtration rate was 79.2 ± 19.1 mL/min/1.73 m^2^, mean low-density lipoprotein cholesterol was 2.57 ± 0.71 mmol/L, and mean left ventricular ejection fraction was 59.3% ± 15.4%. Baseline characteristics are summarized in Table 1.

**Table 1:** Baseline patient characteristics (*n* = 13)

Characteristic	Value
Age (yr)	60.3 ± 9.8
Male sex	11 (84.6)
Body mass index (kg/m^2^)	22.4 ± 3.0
Current or former smoking	9 (69.2)
Diabetes mellitus	5 (38.5)
Hypertension	6 (46.2)
Chronic coronary syndrome	8 (61.5)
NSTEMI	3 (23.1)
Unstable angina	1 (7.7)
Estimated glomerular filtration rate (mL/min/1.73 m^2^)	79.2 ± 19.1
Low-density lipoprotein cholesterol (mmol/L)	2.57 ± 0.71
Left ventricular ejection fraction (%)	59.3 ± 15.4

Values are presented as mean ± standard deviation or number (%)

*NSTEMI* non–ST-segment elevation myocardial infarction

Target bifurcation lesions were distributed in the left anterior descending (LAD)–diagonal bifurcation in nine cases (69.2%), left circumflex–obtuse marginal bifurcation in three cases (23.1%), and posterior descending artery–posterolateral ventricular branch in one case (7.7%). According to Medina classification, lesion distribution comprised 1,1,1 morphology in 12 cases (92.3%) and 0,1,1 morphology in 1 case (7.7%) as summarized in Table 2. Mean bifurcation angle measured 64° ± 28°. No left main bifurcation lesions were included. Target bifurcations were limited to LAD-diagonal, left circumflex–obtuse marginal, and posterior descending artery–posterolateral ventricular anatomies.

**Table 2:** Lesion characteristics (*n* = 13)

Characteristic	No. of cases (%)
Culprit vessel	
Left anterior descending–diagonal bifurcation	9 (69.2)
Left circumflex–obtuse marginal bifurcation	3 (23.1)
Posterior descending artery–posterolateral ventricular branch	1 (7.7)
Bifurcation type	
Medina 1,1,1	12 (92.3)
Medina 0,1,1	1 (7.7)
Medina 1,0,1	0 (0)

### Procedural outcomes

**Fig. 1:**
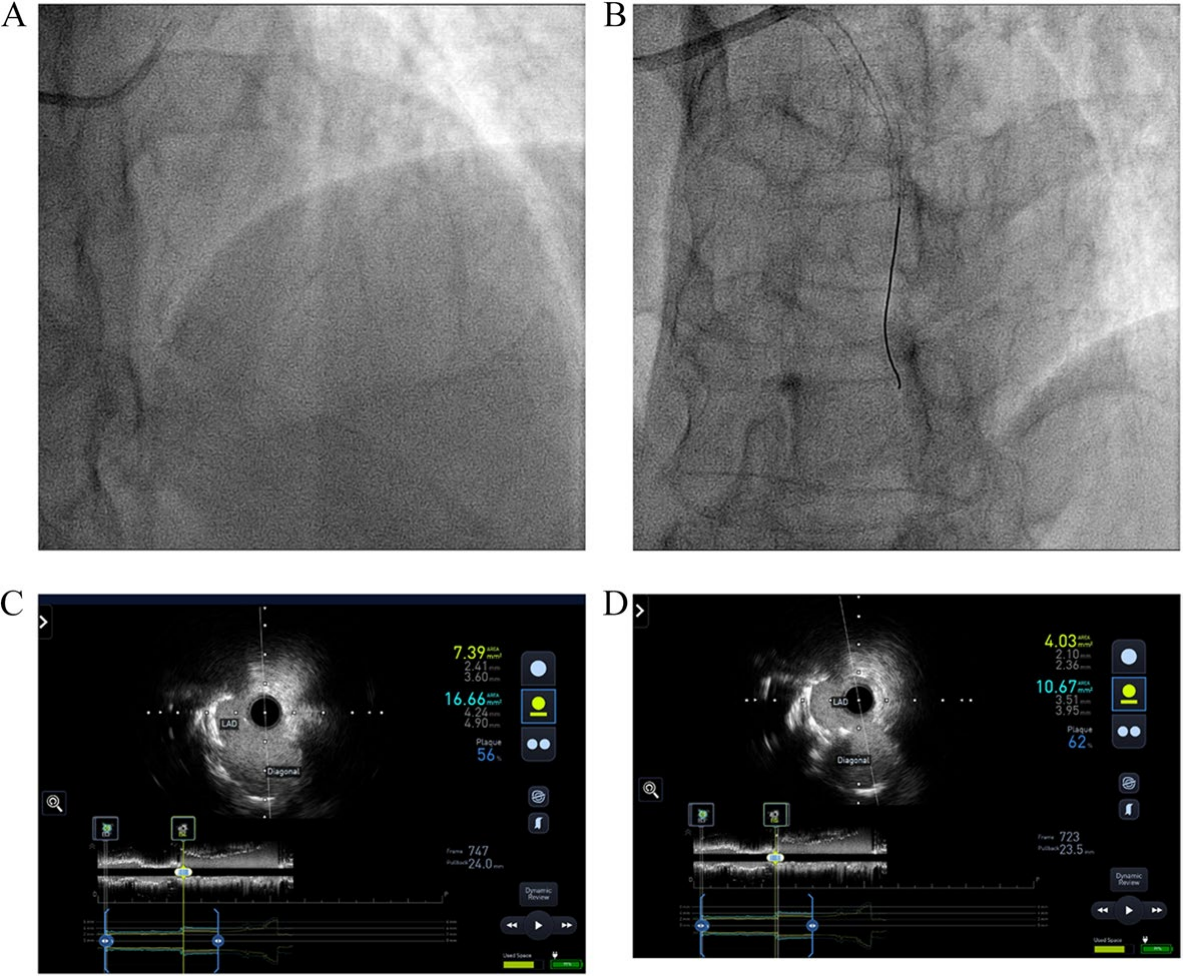
Coronary angiography in a 69-year-old male patient with a history of hypertension and active smoking who presented with exertional left-sided chest pain. Percutaneous coronary angiography demonstrated a severe bifurcation lesion involving the left anterior descending artery and the diagonal branch, with left anterior descending artery dominance. The patient underwent percutaneous coronary intervention using the double kissing culotte technique with two DynamX (Elixir Medical Corp) drug-eluting stents (2.5 × 28 and 2.5 × 33 mm). **A**, **B** Postprocedural digital subtraction angiography showed optimal stent expansion and restoration of coronary flow. **C**, **D** Intravascular ultrasound confirmed adequate stent apposition and full expansion, particularly at the bifurcation segment, with satisfactory coverage of both the main vessel and side branch ostium

**Fig. 2:**
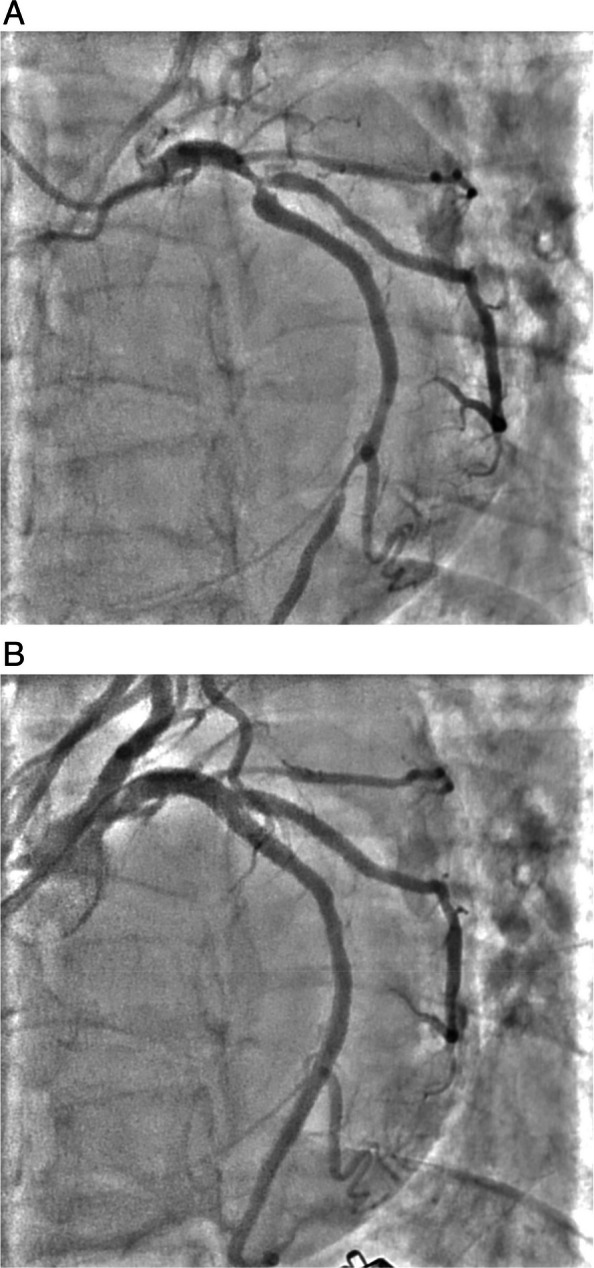
A 72-year-old male patient was admitted to the emergency department with severe angina and was diagnosed with non–ST-segment elevation myocardial infarction. **A**, **B** Transthoracic echocardiography demonstrated a left ventricular ejection fraction of 30%, a left ventricular end-diastolic diameter of 59 mm, and severe functional mitral regurgitation (Carpentier type IIIb). The patient underwent percutaneous coronary intervention with implantation of two DynamX (Elixir Medical Corp) drug-eluting stents (3.5 × 28 and 3.5 × 23 mm) using the culotte technique

**Fig. 3:**
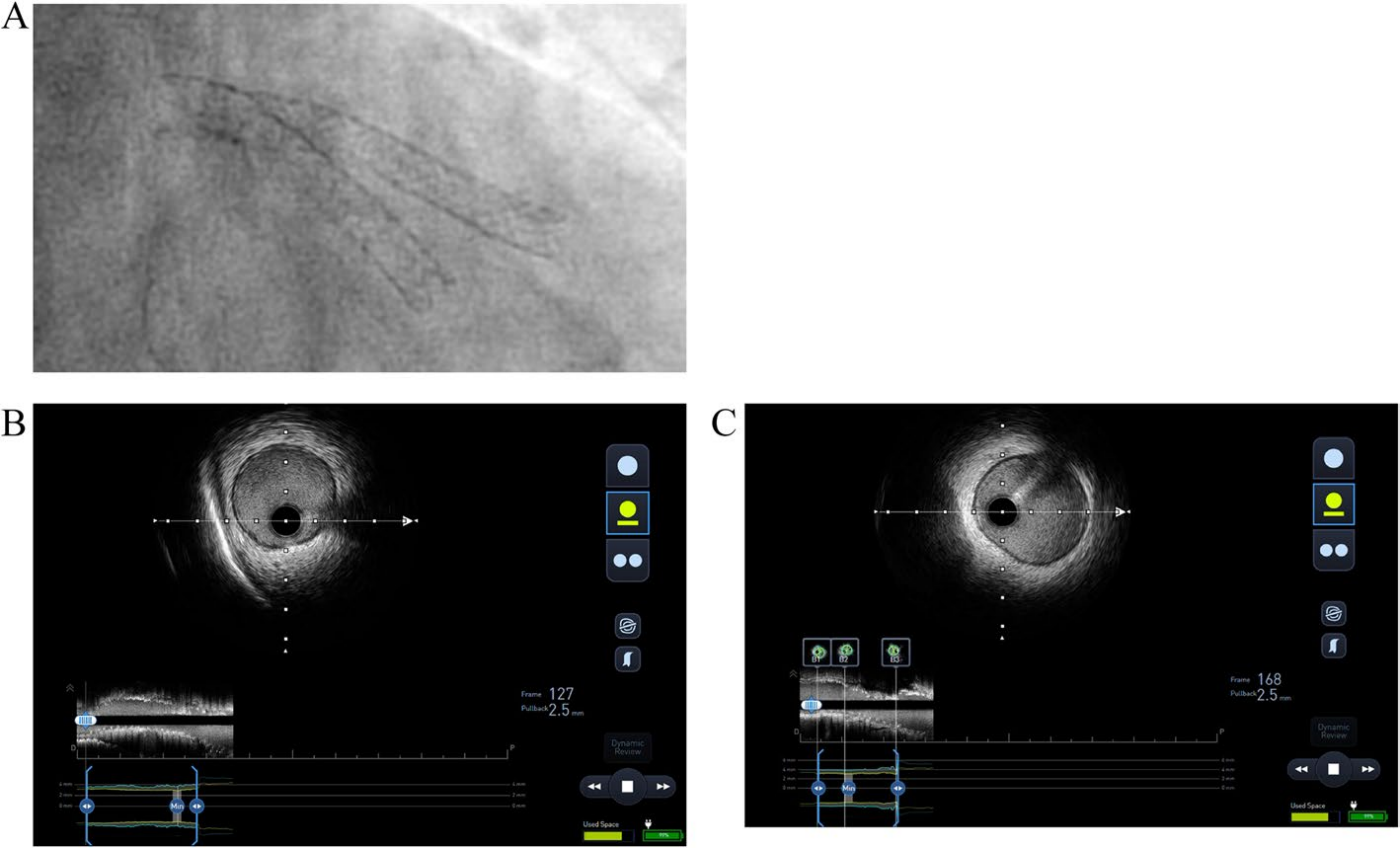
Postprocedural imaging after culotte stenting with two DynamX (Elixir Medical Corp) drug-eluting stents. Intravascular ultrasound (IVUS) assessment of (**B**) the diagonal branch and (**C**) the left anterior descending artery confirmed optimal stent apposition and full expansion, with adequate scaffolding at the bifurcation and no evidence of edge dissection or significant residual stenosis

Technical success was achieved in all 13 patients (100%). All bioadaptor stents were successfully delivered to intended positions, deployed, and adequately expanded according to IVUS criteria. The helical architecture did not create deliverability challenges or impede navigation through guide catheters or tortuous segments in any case (Figs. 1, 2 and 3).

Rewiring through deployed stent struts was accomplished successfully in all cases for both initial (13 of 13, 100%) and subsequent (13 of 13, 100%) rewiring maneuvers. Mean time required for initial rewiring was 39.2 ± 19.0 s (range, 15–75 s), whereas subsequent rewiring required 206.9 ± 162.4 s (range, 45–600 s). Distal cell crossing, the preferred rewiring position, was achieved as intended in all cases without need to accept proximal cell positioning as a compromise. The helical structure with uncaging elements created a cell pattern that operators noted facilitated guidewire crossing.

Final KBI was performed successfully in all cases (13 of 13, 100%), with complete simultaneous balloon expansion without residual waist observed in all procedures. POT was systematically executed at three time points in all cases. Mean procedure time was 55.2 ± 20.7 min, mean fluoroscopy time was 38.4 ± 17.6 min, and mean contrast volume was 218.0 ± 54.9 mL. Procedural outcomes are detailed in Tables 3, 4 and 5.

**Table 3:** Procedural characteristics: devices and technique (patient-level)

Patient No	Main vessel predilatation	Side branch predilatation	Main vessel stent (mm)	Side branch stent (mm)
1	Semi-compliant 2.0, 18 atm	Semi-compliant 2.0, 18 atm	2.5 × 28	2.5 × 33
2	Semi-compliant 2.5, 18 atm	Semi-compliant 2.5, 18 atm	3.5 × 28	3.5 × 23
3	Nonslip element 3.0, 24 atm	Nonslip element 3.0, 24 atm	3.5 × 28	3.0 × 28
4	Not performed	Not performed	3.5 × 28	3.0 × 18
5	Nonslip element 3.0, 20 atm	Not performed	3.0 × 32	3.0 × 18
6	Scoring 2.0, 24 atm	Not performed	2.75 × 23	2.5 × 18
7	Cutting 2.75, 20 atm	Semi-compliant 2.5, 14 atm	3.5 × 23	3.5 × 18
8	Scoring 2.75, 16 atm	Scoring 2.75, 14 atm	3.0 × 38	3.0 × 32
9	Noncompliant 3.5, 14 atm + cutting 2.75, 14 atm	Scoring 2.0, 24 atm	3.5 × 23	3.0 × 23
10	Scoring 3.0, 24 atm	Scoring 3.0, 24 atm	3.5 × 23	3.5 × 32
11	Cutting 2.5, 12 atm	Semi-compliant 2.0, 20 atm	3.0 × 28	2.75 × 23
12	Cutting 2.75, 18 atm	Semi-compliant 2.0, 16 atm	3.5 × 38	3.5 × 18
13	Not performed	Semi-compliant 2.0, 14 atm	3.0 × 38	3.0 × 28

**Table 4:** Procedural characteristics: optimization (patient-level)

Patient No	Main vessel postdilatation	Side branch postdilatation	Final KBI		POT
			Main vessel diameter	Side branch diameter	
1	2.75 mm, 24 atm	2.75 mm, 24 atm	2.5 mm, 10 atm	2.5 mm, 10 atm	3.0 mm, 18 atm
2	3.5 mm, 24 atm	3.25 mm, 20 atm	3.5 mm, 14 atm	3.25 mm, 18 atm	4.0 mm, 16 atm
3	3.5 mm, 24 atm	3.5 mm, 20 atm	3.25 mm, 14 atm	3.0 mm, 14 atm	4.0 mm, 12 atm
4	3.5 mm, 20 atm	3.5 mm, 20 atm	3.25 mm, 16 atm	3.25 mm, 12 atm	4.0 mm, 18 atm
5	3.25 mm, 24 atm	3.0 mm, 24 atm	3.25 mm, 12 atm	3.0 mm, 12 atm	4.0 mm, 12 atm
6	3.25 mm, 24 atm	2.5 mm, 24 atm	2.5 mm, 12 atm	2.5 mm, 12 atm	3.25 mm, 16 atm
7	3.5 mm, 24 atm	3.5 mm, 24 atm	3.25 mm, 16 atm	3.25 mm, 14 atm	4.0 mm, 16 atm
8	3.5 mm, 20 atm	3.5 mm, 14 atm	2.75 mm, 16 atm	2.75 mm, 18 atm	3.5 mm, 18 atm
9	3.5 mm, 12 atm	2.75 mm, 20 atm	3.25 mm, 12 atm	2.75 mm, 12 atm	4.0 mm, 8 atm
10	3.25 mm, 24 atm	3.25 mm, 24 atm	3.25 mm, 14 atm	3.25 mm, 14 atm	4.0 mm, 14 atm
11	2.75 mm, 20 atm	2.75 mm, 18 atm	2.5 mm, 16 atm	2.5 mm, 16 atm	3.25 mm, 22 atm
12	3.5 mm, 24 atm	3.5 mm, 22 atm	3.25 mm, 16 atm	3.0 mm, 16 atm	4.0 mm, 22 atm
13	3.5 mm, 24 atm	3.0 mm, 24 atm	2.75 mm, 16 atm	2.75 mm, 12 atm	4.0 mm, 8 atm

*KBI* kissing balloon inflation

**Table 5:** Overall procedural metrics (*n* = 13)

Variable	Value
Total rewiring time (sec)	246.2 ± 171.8
Contrast volume (mL)	218.0 ± 54.9
Radiation dose (mGy)	1,288.7 ± 666.8
Fluoroscopy time (min)	38.4 ± 17.6

Values are presented as mean ± standard deviation

### IVUS analysis

Comprehensive IVUS imaging with complete quantitative analysis was successfully performed in all 13 patients. In the main branch, mean reference area measured 7.21 ± 1.77 mm^2^ (range, 4.65–9.75 mm^2^), distal stent area measured 7.30 ± 1.88 mm^2^ (range, 4.19–9.49 mm^2^), and overall expansion was 90.9% ± 13.6% (range, 71.3%–123.2%). At the main branch ostium, expected ostial area measured 7.25 ± 1.66 mm^2^ (range, 4.90–9.62 mm^2^), achieved ostial stent area measured 6.83 ± 1.58 mm^2^ (range, 4.57–8.97 mm^2^), and ostial expansion was 94.9% ± 10.6% (range, 81.2%–118.6%) of expected.

In the side branch, mean reference area measured 5.94 ± 1.82 mm^2^ (range, 3.50–9.16 mm^2^), distal stent area measured 6.18 ± 1.55 mm^2^ (range, 3.86–8.82 mm^2^), and overall expansion was 94.5% ± 10.5% (range, 74.6%–114.8%). At the side branch ostium, expected ostial area measured 6.69 ± 1.35 mm^2^ (range, 4.90–8.29 mm^2^), achieved ostial stent area measured 6.03 ± 1.62 mm^2^ (range, 3.16–8.28 mm^2^), and ostial expansion was 90.2% ± 17.5% (range, 61.1%–116.2%) of expected. In the proximal main vessel, proximal stent area measured 10.74 ± 2.53 mm^2^ (range, 6.51–14.89 mm^2^), and proximal expansion ratio (observed to expected) was 0.95 ± 0.16 (range, 0.79–1.20).

IVUS confirmed complete coverage of the carina in all cases, with the characteristic double-layer metal configuration in the proximal main vessel clearly visible. The helical bioadaptor strut pattern demonstrated preserved inter-strut spacing and conformable apposition without distortion. No strut fractures, significant deformations, or malapposition were identified. The unlocking elements and polymer coating appeared intact in all cases. Full IVUS quantitative data are presented in Table 6.

**Table 6:** Quantitative intravascular ultrasound measurements

Parameter	Patient No												
	1	2	3	4	5	6	7	8	9	10	11	12	13
Main branch													
Reference area (mm^2^)	4.65	9.01	8.77	6.29	7.77	5.08	9.75	6.14	6.80	9.53	6.62	8.34	4.99
Distal stent area (mm^2^)	4.19	9.23	9.49	8.58	9.34	4.97	8.43	6.16	7.15	8.17	5.23	8.61	5.40
Expansion (%)	79.1	97.0	71.3	123.2	96.3	87.4	85.5	94.5	103.4	86.4	72.5	94.2	91.4
Minimal stent area (mm^2^)	3.68	8.74	6.25	7.75	7.48	4.44	8.34	5.80	7.03	8.23	4.80	7.86	4.56
Expected ostial area (mm^2^)^a^	4.90	9.62	8.29	8.29	8.29	4.91	8.29	5.94	8.29	8.29	4.91	8.29	5.94
Ostial stent area (mm^2^)	5.81	8.94	6.88	8.97	8.67	4.57	7.17	5.21	8.19	7.53	4.76	6.73	5.41
Ostial expansion (% expected)	118.6	93.0	83.0	108.2	104.6	93.1	86.5	87.8	98.8	90.8	97.0	81.2	91.1
Side branch													
Reference area (mm^2^)	3.80	5.80	5.29	7.35	4.63	3.50	9.16	4.27	5.70	7.55	5.18	8.87	6.16
Distal stent area (mm^2^)	4.08	6.79	5.97	7.44	5.04	3.86	8.82	4.76	6.00	7.96	5.49	7.91	6.28
Expansion (%)	86.3	99.5	83.2	92.9	107.6	97.4	90.7	114.8	100.7	88.1	96.3	74.6	96.6
Minimal stent area (mm^2^)	3.28	5.77	4.40	6.83	4.98	3.41	8.31	4.90	5.74	6.65	4.99	6.62	5.95
Expected ostial area (mm^2^)^a^	4.90	8.29	7.07	8.29	7.07	4.91	8.29	5.94	5.94	8.29	4.91	7.07	5.94
Ostial stent area (mm^2^)	3.90	7.87	4.32	8.21	6.52	3.16	6.04	4.88	6.90	8.28	5.57	6.37	6.32
Ostial expansion (% expected)	79.6	94.9	61.1	99.0	92.3	64.4	72.8	82.2	116.2	99.9	113.5	90.2	106.5
Proximal main branch													
Polygon of confluence area (mm^2^)	8.54	12.64	9.17	15.02	14.91	7.81	11.05	9.17	9.45	14.49	10.04	11.64	10.63
Overlap area (mm^2^)	7.38	13.63	9.70	12.87	13.63	6.99	10.49	7.57	9.62	13.88	8.37	11.98	9.88
Proximal stent area (mm^2^)	7.11	13.84	10.10	11.79	12.37	6.51	12.20	7.79	10.57	14.89	9.95	11.25	10.23
Expected area (mm^2^)^a^	7.07	12.56	12.56	12.56	12.56	8.29	12.56	9.62	12.56	12.56	8.29	12.56	12.56
Expansion (% expected)	1.01	1.10	0.80	0.94	0.98	0.79	0.97	0.81	0.84	1.19	1.20	0.90	0.81

^a^Expected area was derived from the theoretical circular luminal area corresponding to the nominal diameters of the kissing balloons at the bifurcation segments and the re–proximal optimization technique balloon at the proximal main branch segment

### Clinical outcomes

All patients were discharged successfully without in-hospital major adverse cardiac events. No deaths or emergency target vessel revascularization occurred. One patient developed minor access-site hematoma which was managed conservatively. Procedural success was 100% (13 of 13 patients). Four patients demonstrated postprocedural hs-TnT elevation above five times the upper reference limit. However, no patient had new ischemic symptoms, new electrocardiogram changes, angiographic side branch occlusion, or persistent flow-limiting complication after PCI. Because several patients had dynamically rising baseline biomarkers or lacked a stable preprocedural reference value, definitive attribution of biomarker elevation to PCI was not possible. Therefore, no definite periprocedural myocardial infarction was adjudicated, and these cases are reported descriptively (Table 7).

**Table 7:** Clinical scenario for postprocedural hs-TnT elevation cases

Case No	Clinical scenario	Laboratory results
1	PCI was performed on March 28, 2025 at 14:00 Although hs-TnT increased after PCI, the patient had no ischemic symptoms, no new ECG changes, and no angiographic side branch occlusion (≥ 1 mm). In addition, limited preprocedural serial troponin measurements prevented determination of whether the elevation was procedure-related or reflected the natural evolution of myocardial infarction. Therefore, PMI could not be definitively diagnosed	Troponin T: 209 ng/L at 12:35 on March 28 → 399 ng/L at 06:00 on March 29 → 999 ng/L at 11:50 on March 31 → 1,092 ng/L at 06:05 on April 1 → 1,073 ng/L at 14:10 on April 2 CK: 172 U/L at 12:35 on March 28 CK-MB: 18.5 U/L at 12:35 on March 28
2	The patient was admitted with complete atrioventricular block and syncope, and underwent dual-chamber permanent pacemaker implantation on May 28, 2025 PCI was subsequently performed on June 4, 2025 Following PCI, hs-TnT increased; however, the patient had no clinical symptoms suggestive of ischemia, no new ECG changes, and no angiographic side branch occlusion (≥ 1 mm). Additionally, myocardial injury related to recent pacemaker implantation may have contributed to biomarker elevation. Therefore, this case was not classified as definite PMI	Troponin T: 9.7 ng/L at 09:47 on May 28 → 7.8 ng/L at 06:41 on June 4 → 208 ng/L at 07:00 on June 5 CK: 60 U/L at 09:47 on May 28 CK-MB: 15.6 U/L at 09:47 on May 28
3	The patient initially presented with chest pain but declined coronary angiography. On July 17, 2025, recurrent severe chest pain led to admission and PCI; however, no biomarker measurement was obtained immediately prior to the procedure After PCI, cardiac biomarkers were elevated, but the patient’s symptoms improved, with no new ECG changes and no angiographic side branch occlusion (≥ 1 mm). Given the clinical context of non–ST-segment elevation acute coronary syndrome and absence of a reliable preprocedural baseline, attribution of biomarker elevation to PCI was not possible	Troponin T: 18.1 ng/L at 12:06 on July 10 → 936 ng/L at 06:45 on July 18 → 904 ng/L at 07:04 on July 19 CK: 74.6 U/L at 12:06 on July 10 CK-MB: 8.0 U/L at 12:06 on July 10
4	The patient was admitted with NSTEMI and underwent PCI on November 10, 2025, at 07:30 During PCI, slow-flow phenomenon occurred and was treated with adenosine and nitroglycerin, restoring TIMI grade flow 3. However, hs-TnT was already showing a dynamic rising pattern prior to PCI, rather than a stable or falling baseline, precluding strict application of type 4a myocardial infarction. Furthermore, CK-MB demonstrated a decreasing trend after the procedure. Therefore, the biomarker elevation could not be confidently attributed to PCI	Troponin T: 812 ng/L at 03:13 → 893 ng/L at 03:50 on November 10 → 2,647 ng/L at 05:00 on November 11 → 1,710 ng/L at 05:00 on November 12 CK: 1,047 U/L at 03:13 → 1,173 U/L at 03:50 on November 10 CK-MB: 120.6 U/L at 03:13 → 140.1 U/L at 03:50 on November 10 → 84.6 U/L at 05:00 on November 11 → 38.5 U/L at 05:00 on November 12

*CK* creatine kinase, *ECG* electrocardiogram, *hs-TnT* high-sensitivity troponin T, *NSTEMI* non–ST-segment elevation myocardial infarction, *PCI* percutaneous coronary intervention, *PMI* periprocedural myocardial infarction

## Discussion

The present study represents the first systematic evaluation of DynamX bioadaptor feasibility, safety, and acute procedural outcomes in complex bifurcation intervention using the technically demanding culotte technique. Our principal findings demonstrate that the bioadaptor's unique helical architecture and time-dependent uncaging mechanism are fully compatible with the technical and mechanical requirements of two-stent bifurcation techniques, achieving 100% technical success, 100% procedural success, and favorable IVUS-documented acute outcomes across all evaluated parameters.

### Technical feasibility in culotte stenting

Successful rewiring through deployed stent struts represents perhaps the most technically demanding and failure-prone step in culotte stenting, with significant implications for final procedural outcomes [24, 25]. Failure to achieve distal cell crossing can result in incomplete ostial coverage with gap formation, increased stent malapposition at the ostium, and substantially elevated risk of side branch restenosis [26]. In the present series, rewiring through bioadaptor struts was achieved in all cases. This finding should be interpreted cautiously given the small sample size and experienced operator setting. Procedural success was likely facilitated by strict adherence to contemporary culotte principles, including systematic POT, distal cell crossing, sequential strut dilation, final KBI, and final POT. Further clinical studies are required to determine whether these findings are reproducible across larger and more anatomically diverse bifurcation cohorts.

### IVUS assessment

Optimal stent expansion and complete apposition are critical determinants of both acute and long-term outcomes in bifurcation stenting [27, 28]. Optical coherence tomography (OCT) studies have demonstrated that malapposition is particularly common at bifurcation sites, especially in the region of the side branch ostium and the polygon of confluence [29].

Systematic IVUS analysis demonstrated satisfactory expansion across all segments. Mean ostial expansion in the main branch reached 94.9% of expected with 84.6% of lesions achieving ≥ 80% expansion. Side branch ostial expansion (90.2%) showed greater variability, reflecting the technical sensitivity of side branch optimization. Proximal main vessel expansion ratio (0.95) demonstrated adequate conformability despite double-layer metal configuration, with no cases showing marked collapse.

Although overall IVUS-defined expansion was favorable, selected cases demonstrated lower side branch ostial expansion. Suboptimal ostial expansion may result from lesion-specific factors such as the bifurcation angle, eccentric side branch ostial plaque, calcification, vessel size mismatch, or local resistance to balloon expansion despite sequential POT and final KBI. In the cases presented in this study, suboptimal expansion was primarily attributed to the presence of a calcified nodule located opposite the carina at the side branch ostium. Given the limited cohort size, this study was not powered to evaluate correlations between side branch ostial expansion and anatomic variables; however, these observations support the importance of systematic IVUS-guided optimization in culotte bioadaptor implantation.

### Comparison with conventional drug-eluting stent platforms

The performance characteristics of the bioadaptor can be contextualized against conventional drug-eluting stents (DES) used in culotte bifurcation PCI. Its 71-µm strut thickness places it within the ultra-thin strut category, a feature associated with lower restenosis and stent thrombosis rates, particularly relevant in culotte configurations where proximal metal layers double [30, 31]. Contemporary ultra-thin platforms such as Orsiro (Biotronik) and BioMime (Meril Life Sciences) have demonstrated favorable bifurcation outcomes [32], and the bioadaptor platform falls within this advantageous range while uniquely incorporating a mechanism that restores vessel function 6 months after the procedure. The L605 cobalt-chromium alloy permits thin strut design while maintaining radial strength, an important attribute during sequential high-pressure inflations required for POT and KBI [33, 34]. In the present series, IVUS demonstrated consistent expansion without recoil or deformation, supporting the expected strength to thickness advantages of this material platform.

### Potential long-term advantages

Culotte bifurcation PCI creates overlapping implant layers in the proximal main vessel and at the side branch ostium. Although this configuration provides complete ostial and carinal coverage, the increased metal burden and altered strut geometry may disturb local flow, increase wall shear stress heterogeneity, and contribute to delayed healing, restenosis, or late thrombotic risk after conventional dual DES implantation. These concerns are consistent with prior bifurcation and computational flow analyses emphasizing the importance of implant geometry and imaging-guided optimization in bifurcation PCI [35, 36].

The DynamX bioadaptor may offer a mechanistically distinct approach to this limitation. During the early healing phase, it provides DES-like radial support. After approximately 6 months, polymer resorption permits the three helical strands to unlock and separate, enabling dynamic vessel support rather than permanent rigid caging. Prior studies have demonstrated restoration of cyclic pulsatility, compliance, vasomotion, adaptive remodeling, and reduced plaque progression after uncaging [16, 17, 37]. In culotte anatomy, the dynamic structural adaptation with bioadaptor may reduce the long-term hemodynamic disadvantages of overlapping rigid stent layers, normalizing endothelial shear stresses while preserving the established flow lumen by enabling adaptive remodeling.

Beyond demonstrating acute feasibility, the bioadaptor's uncaging mechanism may confer long-term advantages particularly relevant to bifurcation physiology. IVUS studies from the DynamX mechanistic study documented significant increases in vessel area (3%) and device area (5%) at 9 months to 12 months while maintaining lumen area, indicating positive adaptive remodeling [38].

The BIOADAPTOR RCT trial provided objective imaging evidence that following polymer resorption at approximately 6 months, lumen area increased 7.5% between systole and diastole, approximating untreated vessels, whereas conventional DES remained rigid [37]. In bifurcations, where flow dynamics are inherently complex with varying shear stress distribution, restoration of physiologic pulsatility might favorably modulate these adverse flow patterns [39].

This adaptive capacity could prove particularly beneficial at bifurcation sites, where conventional permanently caged stents prevent natural vessel adaptation and may contribute to late lumen loss and neoatherosclerosis [40]. Although culotte technique inevitably creates double-layer metal in the proximal segment, the bioadaptor’s mechanism theoretically permits partial strand separation after 6 months, potentially reducing effective metal burden compared with permanently overlapped conventional stents [41]. The ADAPT-CULOTTE case report demonstrated favorable 15-month outcomes, with angiography and OCT confirming excellent patency without neoatherosclerosis and restoration of physiologic pulsatility at the left main bifurcation [22]. This systematic study confirms the acute findings of the previous ADAPT-CULOTTE publications in a larger cohort.

Emerging randomized data from two global multicenter trials support the clinical relevance of bioadaptor's mechanism. In the BIOADAPTOR RCT study, the 2-year follow-up showed lower TLF with the bioadaptor than with contemporary DES (1.8% vs. 5.5%, *P* = 0.044), lower target vessel failure (1.8% vs. 5.9%, *P* = 0.027), and no definite or probable device thrombosis in the bioadaptor arm [18]. At 3 years, the BIOADAPTOR RCT trial further demonstrated sustained benefit, including significantly lower TLF in LAD lesions treated with bioadaptor versus DES (2.7% vs. 10.6%; HR, 0.23; 95% CI, 0.06–0.85; *P* = 0.019) and in proximal LAD lesions (2.8% vs. 16.8%, *P* = 0.045) [42]. The large-scale INFINITY-SWEDEHEART randomized controlled trial demonstrated a 48% reduction in TLF from 6 months through 2 years in favor of bioadaptor (HR, 0.52; 95% CI, 0.29–0.93; *P* = 0.027), with significant reduction in target vessel failure and sustained benefit in the acute coronary syndrome subgroup [19]. Together, these data support the concept that the benefit of the bioadaptor may become more apparent after the 6-month unlocking period, when vessel function and hemodynamic modulation begin to recover.

Left main bifurcations were not included in this cohort. While the bioadaptor's ability to provide dynamic support and adaptive remodeling may be attractive in left main bifurcation anatomy, left main PCI involves larger vessel dimensions, greater myocardial territory at risk, and more complex sizing and optimization requirements. Therefore, dedicated studies with long-term imaging and clinical follow-up are needed to evaluate clinical benefit of bioadaptor in left main disease.

### Limitations

This study was limited by its small sample size, single-center design, absence of a control group, and short-term follow-up. Only acute IVUS outcomes were assessed; therefore, the proposed post-uncaging hemodynamic benefits in culotte bifurcation anatomy remain hypothesis-generating. In addition, left main bifurcations were not included, limiting generalizability to this higher-risk anatomical subset. The single-center setting reflects experienced operator expertise, and predefined exclusion criteria further restrict applicability across the broader range of bifurcation anatomies encountered in routine practice. While IVUS was used, OCT would provide higher-resolution strut-level assessment.

## Conclusions

Early experience supports that bioadaptor implantation is feasible for culotte bifurcation stenting, with favorable acute IVUS findings in selected patients. The ultra-thin (71 µm) cobalt-chromium helical architecture with time-dependent uncaging did not impede execution of this complex two-stent technique, achieving 100% technical and procedural success, complete rewiring with optimal distal cell crossing, successful final KBI, and favorable acute IVUS outcomes with satisfactory expansion and apposition across all bifurcation segments. These findings support further investigation in larger, multicenter studies comparing bioadaptive and other PCI platforms, with emphasis on long-term clinical and physiological outcomes.

## Data Availability

Datasets generated during and/or analyzed during the current study are publicly available, available upon reasonable request.

## Abbreviations

CI: Confidence interval
CK: Creatine kinase
DES: Drug-eluting stents
HR: Hazard ratio
hs-TnT: High-sensitivity troponin T
IVUS: Intravascular ultrasound
KBI: Kissing balloon inflation
LAD: Left anterior descending
OCT: Optical coherence tomography
PCI: Percutaneous coronary intervention
POT: Proximal optimization technique
TLF: Target lesion failure

## References

[1] Hildick-Smith D, Lassen JF, Albiero R, Lefevre T, Darremont O, Pan M, et al. Consensus from the 5th European Bifurcation Club meeting. EuroIntervention. 2010;6:34–8. 20542795

[2] Lassen JF, Holm NR, Stankovic G, Lefèvre T, Chieffo A, Hildick-Smith D, et al. Percutaneous coronary intervention for coronary bifurcation disease: consensus from the first 10 years of the European Bifurcation Club meetings. EuroIntervention. 2014;10:545–60. 25256198 10.4244/EIJV10I5A97

[3] Sawaya FJ, Lefèvre T, Chevalier B, Garot P, Hovasse T, Morice MC, et al. Contemporary approach to coronary bifurcation lesion treatment. JACC Cardiovasc Interv. 2016;9:1861–78. 27659563 10.1016/j.jcin.2016.06.056

[4] Behan MW, Holm NR, Curzen NP, Erglis A, Stables RH, de Belder AJ, et al. Simple or complex stenting for bifurcation coronary lesions: a patient-level pooled-analysis of the Nordic Bifurcation Study and the British Bifurcation Coronary Study. Circ Cardiovasc Interv. 2011;4:57–64. 21205942 10.1161/CIRCINTERVENTIONS.110.958512

[5] Lassen JF, Burzotta F, Banning AP, Lefèvre T, Darremont O, Hildick-Smith D, et al. Percutaneous coronary intervention for the left main stem and other bifurcation lesions: 12th consensus document from the European Bifurcation Club. EuroIntervention. 2018;13:1540–53. 29061550 10.4244/EIJ-D-17-00622

[6] Adriaenssens T, Byrne RA, Dibra A, Iijima R, Mehilli J, Bruskina O, et al. Culotte stenting technique in coronary bifurcation disease: angiographic follow-up using dedicated quantitative coronary angiographic analysis and 12-month clinical outcomes. Eur Heart J. 2008;29:2868–76. 19001472 10.1093/eurheartj/ehn512

[7] Chevalier B, Glatt B, Royer T, Guyon P. Placement of coronary stents in bifurcation lesions by the “culotte” technique. Am J Cardiol. 1998;82:943–9. 9794349 10.1016/s0002-9149(98)00510-4

[8] Burzotta F, Lassen JF, Lefèvre T, Banning AP, Chatzizisis YS, Johnson TW, et al. Percutaneous coronary intervention for bifurcation coronary lesions: the 15th consensus document from the European Bifurcation Club. EuroIntervention. 2021;16:1307–17. 33074152 10.4244/EIJ-D-20-00169 PMC8919527

[9] Chen SL, Sheiban I, Xu B, Jepson N, Paiboon C, Zhang JJ, et al. Impact of the complexity of bifurcation lesions treated with drug-eluting stents: the DEFINITION study (Definitions and impact of complEx biFurcation lesIons on clinical outcomes after percutaNeous coronary IntervenTIOn using drug-eluting steNts). JACC Cardiovasc Interv. 2014;7:1266–76. 25326748 10.1016/j.jcin.2014.04.026

[10] Kolandaivelu K, Swaminathan R, Gibson WJ, Kolachalama VB, Nguyen-Ehrenreich KL, Giddings VL, et al. Stent thrombogenicity early in high-risk interventional settings is driven by stent design and deployment and protected by polymer-drug coatings. Circulation. 2011;123:1400–9. 21422389 10.1161/CIRCULATIONAHA.110.003210 PMC3131199

[11] Ormiston JA, Dixon SR, Webster MW, Ruygrok PN, Stewart JT, Minchington I, et al. Stent longitudinal flexibility: a comparison of 13 stent designs before and after balloon expansion. Catheter Cardiovasc Interv. 2000;50:120–4. 10816296 10.1002/(sici)1522-726x(200005)50:1<120::aid-ccd26>3.0.co;2-t

[12] Foin N, Torii R, Mortier P, De Beule M, Viceconte N, Chan PH, et al. Kissing balloon or sequential dilation of the side branch and main vessel for provisional stenting of bifurcations: lessons from micro-computed tomography and computational simulations. JACC Cardiovasc Interv. 2012;5:47–56. 22230150 10.1016/j.jcin.2011.08.019

[13] Mortier P, Hikichi Y, Foin N, De Santis G, Segers P, Verhegghe B, et al. Provisional stenting of coronary bifurcations: insights into final kissing balloon post-dilation and stent design by computational modeling. JACC Cardiovasc Interv. 2014;7:325–33. 24650404 10.1016/j.jcin.2013.09.012

[14] Ormiston JA, Webber B, Ubod B, White J, Webster MW. Coronary stent durability and fracture: an independent bench comparison of six contemporary designs using a repetitive bend test. EuroIntervention. 2015;10:1449–55. 25420788 10.4244/EIJY14M11_08

[15] Kandzari DE, Koolen JJ, Doros G, Massaro JJ, Garcia-Garcia HM, Bennett J, et al. Ultrathin bioresorbable polymer sirolimus-eluting stents versus thin durable polymer everolimus-eluting stents. J Am Coll Cardiol. 2018;72:3287–97. 30257191 10.1016/j.jacc.2018.09.019

[16] Verheye S, Vrolix M, Montorfano M, Zivelonghi C, Giannini F, Bedogni F, et al. Twelve-month clinical and imaging outcomes of the uncaging coronary DynamX bioadaptor system. EuroIntervention. 2020;16:e974–81. 32894231 10.4244/EIJ-D-20-00763

[17] Saito S, Bennett J, Nef HM, Webster M, Namiki A, Takahashi A, et al. First randomised controlled trial comparing the sirolimus-eluting bioadaptor with the zotarolimus-eluting drug-eluting stent in patients with *de novo* coronary artery lesions: 12-month clinical and imaging data from the multi-centre, international, BIODAPTOR-RCT. EClinicalMedicine. 2023;65:102304. 38106564 10.1016/j.eclinm.2023.102304 PMC10725075

[18] Saito S, Bennett J, Nef HM, Webster M, Namiki A, Takahashi A, et al. Percutaneous coronary treatment with bioadaptor implant vs drug-eluting stent: 2-year outcomes from BIOADAPTOR RCT. JACC Cardiovasc Interv. 2025;18:988–97. 40057888 10.1016/j.jcin.2025.01.426

[19] Erlinge D, Andersson J, Fröbert O, Törnerud M, Hamid M, Kellerth T, et al. Bioadaptor implant versus contemporary drug-eluting stent in percutaneous coronary interventions in Sweden (INFINITY-SWEDEHEART): a single-blind, non-inferiority, registry-based, randomised controlled trial. Lancet. 2024;404:1750–9. 39481425 10.1016/S0140-6736(24)02227-X

[20] Nakazawa G, Otsuka F, Nakano M, Vorpahl M, Yazdani SK, Ladich E, et al. The pathology of neoatherosclerosis in human coronary implants bare-metal and drug-eluting stents. J Am Coll Cardiol. 2011;57:1314–22. 21376502 10.1016/j.jacc.2011.01.011 PMC3093310

[21] Otsuka F, Byrne RA, Yahagi K, Mori H, Ladich E, Fowler DR, et al. Neoatherosclerosis: overview of histopathologic findings and implications for intravascular imaging assessment. Eur Heart J. 2015;36:2147–59. 25994755 10.1093/eurheartj/ehv205

[22] Wong SF, Chow HC, Chan K, Chung TS. The double bioadaptors culotte (ADAPT-CULOTTE) technique: from bench testing to the first-in-human longitudinal imaging analysis. JACC Cardiovasc Interv. 2024;17:2957–60. 39466212 10.1016/j.jcin.2024.09.059

[23] Thygesen K, Alpert JS, Jaffe AS, Chaitman BR, Bax JJ, Morrow DA, et al. Fourth universal definition of myocardial infarction (2018). J Am Coll Cardiol. 2018;72:2231–64. 30153967 10.1016/j.jacc.2018.08.1038

[24] Foin N, Sen S, Allegria E, Petraco R, Nijjer S, Francis DP, et al. Maximal expansion capacity with current DES platforms: a critical factor for stent selection in the treatment of left main bifurcations? EuroIntervention. 2013;8:1315–25. 23086760 10.4244/EIJV8I11A200

[25] Murasato Y, Finet G, Foin N. Final kissing balloon inflation: the whole story. EuroIntervention. 2015;11(Suppl V):V81–5. 25983179 10.4244/EIJV11SVA18

[26] Kang SJ, Mintz GS, Kim WJ, Lee JY, Oh JH, Park DW, et al. Changes in left main bifurcation geometry after a single-stent crossover technique: an intravascular ultrasound study using direct imaging of both the left anterior descending and the left circumflex coronary arteries before and after intervention. Circ Cardiovasc Interv. 2011;4:355–61. 21712525 10.1161/CIRCINTERVENTIONS.110.961045

[27] Gutiérrez-Chico JL, Wykrzykowska J, Nüesch E, van Geuns RJ, Koch KT, Koolen JJ, et al. Vascular tissue reaction to acute malapposition in human coronary arteries: sequential assessment with optical coherence tomography. Circ Cardiovasc Interv. 2012;5:20–9. 22319063 10.1161/CIRCINTERVENTIONS.111.965301

[28] Räber L, Mintz GS, Koskinas KC, Johnson TW, Holm NR, Onuma Y, et al. Clinical use of intracoronary imaging. Part 1: guidance and optimization of coronary interventions. An expert consensus document of the European Association of Percutaneous Cardiovascular Interventions. Eur Heart J. 2018;39:3281–300. 10.1093/eurheartj/ehy285 29790954

[29] Okamura T, Onuma Y, Yamada J, Iqbal J, Tateishi H, Nao T, et al. 3D optical coherence tomography: new insights into the process of optimal rewiring of side branches during bifurcational stenting. EuroIntervention. 2014;10:907–15. 24531393 10.4244/EIJV10I8A157

[30] Bangalore S, Toklu B, Amoroso N, Fusaro M, Kumar S, Hannan EL, et al. Bare metal stents, durable polymer drug eluting stents, and biodegradable polymer drug eluting stents for coronary artery disease: mixed treatment comparison meta-analysis. BMJ. 2013;347:f6625. 24212107 10.1136/bmj.f6625 PMC3898413

[31] Kastrati A, Mehilli J, Dirschinger J, Dotzer F, Schühlen H, Neumann FJ, et al. Intracoronary stenting and angiographic results: strut thickness effect on restenosis outcome (ISAR-STEREO) trial. Circulation. 2001;103:2816–21. 11401938 10.1161/01.cir.103.23.2816

[32] Naber CK, Urban P, Ong PJ, Valdes-Chavarri M, Abizaid AA, Pocock SJ, et al. Biolimus-A9 polymer-free coated stent in high bleeding risk patients with acute coronary syndrome: a Leaders Free ACS sub-study. Eur Heart J. 2017;38:961–9. 27190095 10.1093/eurheartj/ehw203 PMC5837685

[33] Hermawan H, Dubé D, Mantovani D. Developments in metallic biodegradable stents. Acta Biomater. 2010;6:1693–7. 19815097 10.1016/j.actbio.2009.10.006

[34] O’Brien B, Carroll W. The evolution of cardiovascular stent materials and surfaces in response to clinical drivers: a review. Acta Biomater. 2009;5:945–58. 19111513 10.1016/j.actbio.2008.11.012

[35] Katritsis DG, Theodorakakos A, Pantos I, Gavaises M, Karcanias N, Efstathopoulos EP. Flow patterns at stented coronary bifurcations: computational fluid dynamics analysis. Circ Cardiovasc Interv. 2012;5:530–9. 22763345 10.1161/CIRCINTERVENTIONS.112.968347

[36] Park SJ, Kim YH, Park DW, Lee SW, Kim WJ, Suh J, et al. Impact of intravascular ultrasound guidance on long-term mortality in stenting for unprotected left main coronary artery stenosis. Circ Cardiovasc Interv. 2009;2:167–77. 20031713 10.1161/CIRCINTERVENTIONS.108.799494

[37] Verheye S, Morice MC, Zivelonghi C, Mehmedbegovic Z, Neylon A, Bhat V, et al. 24-Month clinical follow-up and mechanistic insights from intravascular imaging following coronary implantation of the novel DynamX bioadaptor platform. Cardiovasc Revasc Med. 2023;46:106–12. 36184491 10.1016/j.carrev.2022.09.009

[38] Chatzizisis YS, Coskun AU, Jonas M, Edelman ER, Feldman CL, Stone PH, et al. Role of endothelial shear stress in the natural history of coronary atherosclerosis and vascular remodeling: molecular, cellular, and vascular behavior. J Am Coll Cardiol. 2007;49:2379–93. 17599600 10.1016/j.jacc.2007.02.059

[39] Webster M, Scott D, Menon M, McClean D, El-Jack S, Wilkins G, et al. Percutaneous coronary intervention using the DynamX sirolimus-eluting bioadaptor: 12-month clinical and imaging outcomes. J Interv Cardiol. 2024;2024:8876443.

[40] Torii S, Jinnouchi H, Sakamoto A, Kutyna M, Cornelissen A, Kuntz S, et al. Drug-eluting coronary stents: insights from preclinical and pathology studies. Nat Rev Cardiol. 2020;17:37–51. 31346257 10.1038/s41569-019-0234-x

[41] Joner M, Finn AV, Farb A, Mont EK, Kolodgie FD, Ladich E, et al. Pathology of drug-eluting stents in humans: delayed healing and late thrombotic risk. J Am Coll Cardiol. 2006;48:193–202. 16814667 10.1016/j.jacc.2006.03.042

[42] Bennett JR. Three year results in patients with LAD lesions treated with DynamX bioadaptor compared to a contemporary DES: from the BIOADAPTOR-RCT trial. Transcatheter Cardiovascular Therapeutics (TCT) 2025; 2025 Oct 25–28; San Francisco, CA, USA.

